# The evolutionary consequences of interactions between the epigenome, the genome and the environment

**DOI:** 10.1111/eva.13730

**Published:** 2024-07-23

**Authors:** Pierre Baduel, Iris Sammarco, Rowan Barrett, Marta Coronado‐Zamora, Amélie Crespel, Bárbara Díez‐Rodríguez, Janay Fox, Dario Galanti, Josefa González, Alexander Jueterbock, Eric Wootton, Ewan Harney

**Affiliations:** ^1^ Institut de Biologie de l'Ecole Normale Supérieure PSL University, CNRS Paris France; ^2^ Institute of Botany of the Czech Academy of Sciences Průhonice Czechia; ^3^ Redpath Museum and Department of Biology McGill University Montreal Canada; ^4^ Institute of Evolutionary Biology CSIC, UPF Barcelona Spain; ^5^ Department of Biology University of Turku Turku Finland; ^6^ Natural Resources and Climate Area CARTIF Technology Centre Valladolid Spain; ^7^ Institute of Evolution and Ecology (EvE) University of Tuebingen Tübingen Germany; ^8^ Algal and Microbial Biotechnology Division, Faculty of Biosciences and Aquaculture Nord University Bodø Norway; ^9^ School of Biosciences University of Sheffield Sheffield UK

**Keywords:** DNA methylation, epigenetics, gene–environment interactions, natural populations, transgenerational effects, transposable elements

## Abstract

The epigenome is the suite of interacting chemical marks and molecules that helps to shape patterns of development, phenotypic plasticity and gene regulation, in part due to its responsiveness to environmental stimuli. There is increasing interest in understanding the functional and evolutionary importance of this sensitivity under ecologically realistic conditions. Observations that epigenetic variation abounds in natural populations have prompted speculation that it may facilitate evolutionary responses to rapid environmental perturbations, such as those occurring under climate change. A frequent point of contention is whether epigenetic variants reflect genetic variation or are independent of it. The genome and epigenome often appear tightly linked and interdependent. While many epigenetic changes are genetically determined, the converse is also true, with DNA sequence changes influenced by the presence of epigenetic marks. Understanding how the epigenome, genome and environment interact with one another is therefore an essential step in explaining the broader evolutionary consequences of epigenomic variation. Drawing on results from experimental and comparative studies carried out in diverse plant and animal species, we synthesize our current understanding of how these factors interact to shape phenotypic variation in natural populations, with a focus on identifying similarities and differences between taxonomic groups. We describe the main components of the epigenome and how they vary within and between taxa. We review how variation in the epigenome interacts with genetic features and environmental determinants, with a focus on the role of transposable elements (TEs) in integrating the epigenome, genome and environment. And we look at recent studies investigating the functional and evolutionary consequences of these interactions. Although epigenetic differentiation in nature is likely often a result of drift or selection on stochastic epimutations, there is growing evidence that a significant fraction of it can be stably inherited and could therefore contribute to evolution independently of genetic change.

## 1 INTRODUCTION

The epigenome refers to the suite of epigenetic marks, modifications and molecules that modify the expression and structure of the DNA without altering its underlying sequence. It plays a variety of roles across the tree of life, especially in eukaryotes, influencing development (Feng et al., 2010), phenotypic plasticity (Zhang et al., 2013) and gene regulation (Taudt et al., 2016). The epigenome displays sensitivity to the environment in some cases but is also stable enough to be transgenerationally inherited in others, making it a strong candidate for linking environmental change with evolutionary processes (Fitz‐James & Cavalli, 2022). Recent interest in the relevance of epigenome variation in the wild has confirmed that epigenetic variation is common in natural populations under ecologically relevant conditions (Husby, 2022).

Our understanding of epigenetic mechanisms in model organisms has improved considerably in the last few decades (Bird, 2007; Cavalli & Heard, 2019). However, in natural populations, it remains contentious to what extent the epigenome is independent of the genome or simply represents an extension of the genetic machinery. Furthermore, it is often difficult to disentangle interactions between the genome and epigenome in variable environments, complicating our ability to draw conclusions about their evolutionary importance. Recently, there has been increased interest in exploring how the genome, epigenome and environment interact to shape natural population variation (De Kort et al., 2020; Heckwolf et al., 2020). This is in part driven by recent technological advances that allow us to expand beyond studying specific strains and model organisms and address the role of the epigenome in non‐model organisms and natural populations. This problem is of particular interest to evolutionary biologists and ecologists because if the environmentally determined component of the epigenome can be stably transmitted across generations, it could provide a mechanism for genetic assimilation, whereby selection for non‐genetic changes leads to phenotypic evolution (Nishikawa & Kinjo, 2018; Waddington, 1942). This in turn could facilitate rapid adaptation to global change (McGuigan et al., 2021).

This review provides an overview of recent findings intersecting the fields of ecology, evolution and epigenetics in eukaryotes, with a focus on the plant and animal kingdoms. We outline: (i) the main components of the epigenome, how they vary within and between taxa, and how their transmission fidelity varies; (ii) how variation in the epigenome, especially DNA methylation, interacts with genetic features and environmental determinants, with particular attention on the role of transposable elements (TEs) in integrating the epigenome, genome and environment; and (iii) the functional and evolutionary implications of these interactions. We purposefully draw on findings from a broad range of animal and plant taxa to identify commonalities and distinguish derived features of the epigenome. Finally, we attempt to synthesize key points that have emerged from this rapidly growing field, drawing conclusions about the contexts in which epigenetics is most likely to affect the evolution of natural populations and suggesting approaches that may expand our knowledge further.

## 2 COMPOSITION, SETTING AND RESETTING OF THE EPIGENOME

The epigenome consists of numerous different molecular marks and modifiers, including DNA methylation, histone modifications, non‐coding RNAs (ncRNAs) and patterns of chromatin accessibility and nucleosome occupancy, all of which play a role in the regulation of gene expression. DNA methylation is generally considered the most stable and heritable (Lämke & Bäurle, 2017) and, among eukaryotes, it is primarily found in the form of 5‐methylcytosine (5mC) occurring in all sequence contexts: cytosine guanine dinucleotides (herein labelled CpGs, but also referred to as CG in the plant literature), CHG and CHH (where H is every base except for G). DNA methylation in all three sequence contexts is frequent in plants; however, among animals, CHG and CHH methylation is far less common. In most metazoans, CpG methylation predominates (Gallego‐Bartolomé, 2020), although mammalian mitochondrial DNA does also show non‐CpG methylation (Bellizzi et al., 2013; Patil et al., 2019), and evidence is emerging of a role of non‐CpG methylation in the evolution of vertebrate neural tissue (de Mendoza et al., 2021). Although this review will focus on 5mC, other types of methylation can also occur, which we describe briefly in Box 1.

BOX 1 **Alternative forms of DNA methylation**

Although most frequently DNA methylation refers to 5mC, other forms of methylation, including N6‐adenine methylation (6 mA), 4‐methylcytosine (4mC) and DNA hydroxymethylation, play different roles across the tree of life. 6 mA has been reported in the genomes of bacteria and eukaryotes, fulfilling a diverse array of biological functions (Boulias & Greer, 2022; O'Brown & Greer, 2022). However, its existence in multicellular eukaryotes is controversial, occurring rarely and/or under specific circumstances such as hypoxia or electron transport chain stress (O'Brown & Greer, 2022). In fungi, a negative correlation between methyltransferases associated with 5mC and 6mA could suggest an epigenomic conflict between these two regulatory pathways (Bewick et al., 2019). In bacteria, 4mC is involved in restriction‐modification (RM) systems that protect the genome from foreign DNA, as well as in the regulation of gene expression and DNA replication (Seong et al., 2021). While 4mC is present in diverse protists, such as ciliates, dinoflagellates and apicomplexans (Varma et al., 2022), its distribution and role in eukaryotes remain poorly understood. Finally, DNA hydroxymethylation is found in many organisms. In bacteria, it is involved in DNA restriction and modification systems. In plants, it is involved in gene silencing, stress response and development, while in animals, it is mainly present in the central nervous system, where it regulates neuronal differentiation, plasticity and memory formation (Nasrullah et al., 2022).

**FIGURE 1:**
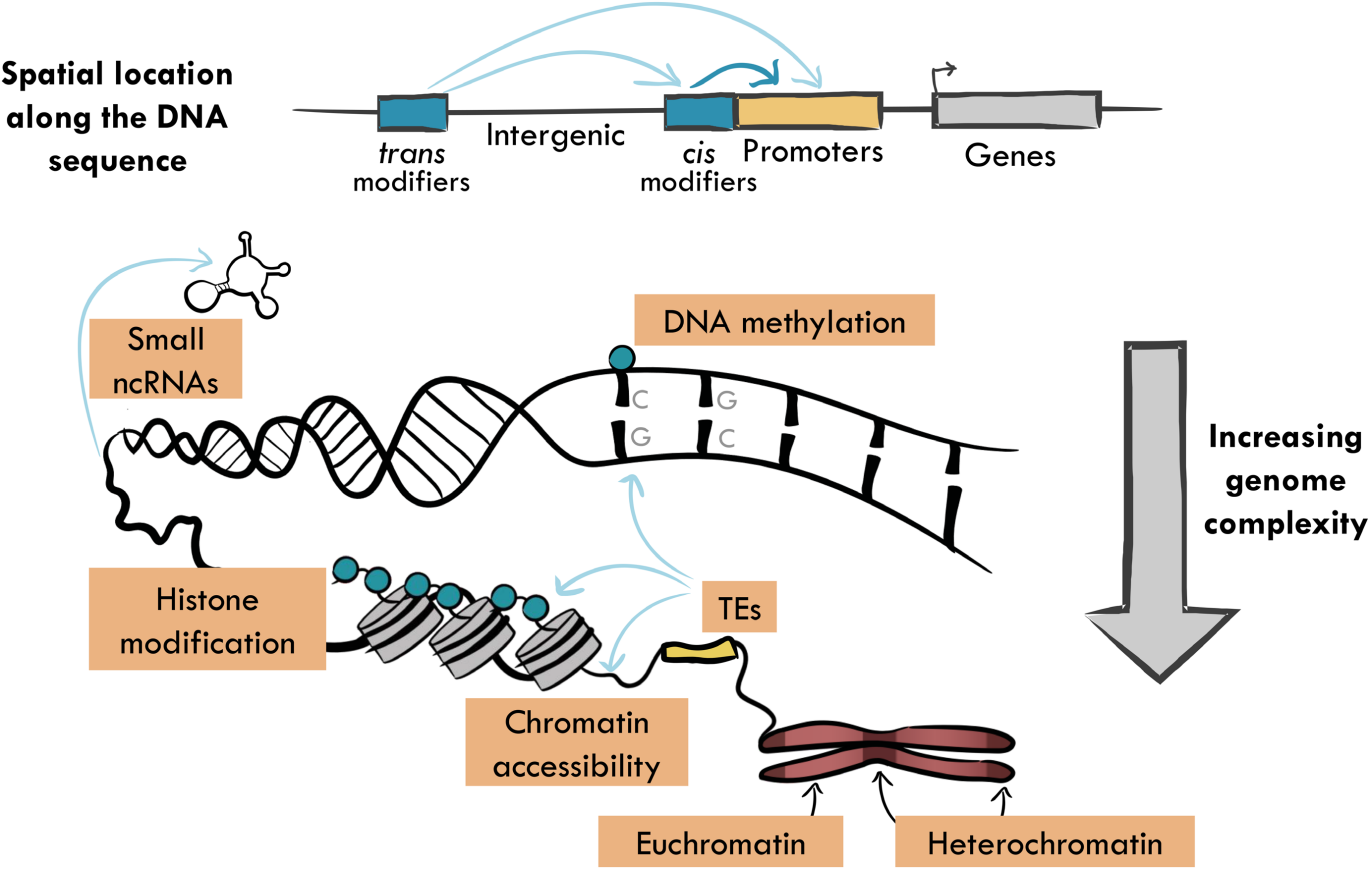
Epigenomic variation occurs across a range of spatial scales within the genome. Its effect depends on proximity to different genomic elements: trans modifiers include DNA methyltransferases, while cis modifiers are often associated with the presence of novel TE insertions. The effects of DNA methylation (blue circles) depend on whether it occurs in regulatory elements, promoters or gene bodies. Distinct mechanisms operate at different hierarchical and organizational levels within the genome, from the expression of ncRNAs to histone modifications and changes in chromatin accessibility. These different mechanisms interact with one another and are influenced by genomic factors such as TE insertions. The presence of many repressive epigenetic marks is associated with the formation of heterochromatin, while more active epigenetic marks are associated with euchromatin formation. Epigenomic variation is also temporally variable; epigenetic effects may be dynamic within a single generation or stably transmitted across one or many generations.

Although DNA methylation is the most frequently studied element of the epigenome, other epigenetic factors, such as histone modifications and ncRNAs, can also influence gene regulation (Figure 1). Histone modifications include distinct types of methylation, phosphorylation and acetylation, which alter the accessibility of the chromatin (Bannister & Kouzarides, 2011), while ncRNAs are often transmitted from parents to offspring (Wang et al., 2017) and contribute to the phenomenon of parental effects and transgenerational epigenetic inheritance. These interacting and interdependent epigenetic factors and their effects on gene regulation vary in importance depending on where they occur in the genome, but together form an integrated system of inherited gene regulation (Adrian‐Kalchhauser et al., 2020) that interacts with the genome to shape phenotypic development. Their presence and importance also vary substantially between taxonomic groups, leading to a complex and variable landscape of epigenetic marks. Here we describe how these epigenetic mechanisms vary within and between genomes from different populations and taxa and provide an overview of the fidelity with which they are transmitted across generations.

### 2.1 Epigenetic landscapes across the genome

Epigenetic marks are not uniformly distributed across genomes. Within the chromosomes of most eukaryotes, there are regions where chromatin is highly compacted (heterochromatin) and others where it is more loosely organized (euchromatin). Heterochromatic regions are typically characterized by a high density of repressive marks and transposable element sequences (TEs), which are usually concentrated within centromeres and telomeres. In contrast, euchromatic regions, typically found within chromosome arms, harbour the large majority of genes and transcriptionally active regions of the genome and display more complex epigenetic patterns with both repressive and permissive marks. The distribution of euchromatin and heterochromatin is highly variable, and significant differences in their relative proportions occur even between closely related species, as seen, e.g. in comparisons of different *Arabidopsis* (Seymour et al., 2014) and *Drosophila* (Marchetti et al., 2022) species.

Genome‐wide DNA methylation levels also vary considerably across genomic features (Figure 1), i.e. TEs, promoters and gene bodies, among species (for a review, see Ritter & Niederhuth, 2021), and in plants, this is the case across all sequence contexts (CpG, CHG and CHH) (Niederhuth et al., 2016). TE sequences are usually heavily methylated and targeted by repressive histone modifications, with both epigenetic marks playing a key role in controlling the mobilization of mutagenic TE copies (Baduel & Quadrana, 2021). Selective methylation of TEs is more widespread in plants and vertebrates than in unicellular animals and fungi (Zemach et al., 2010), suggesting that it is a common mechanism for controlling TE mobilization and highlighting the fundamental role of DNA methylation in genomic stability and evolution. In contrast, DNA methylation of promoters and regulatory regions, although widespread in both plants and vertebrates, presents a more complex picture. Promoter methylation appears to be absent in molluscs (Fallet et al., 2020) and uncommon in arthropods (Lewis et al., 2020), although its presence in centipedes, mealybugs (Lewis et al., 2020) and two distinct sponge species (de Mendoza et al., 2019) suggests multiple evolutionary origins in metazoans. As with the methylation of TEs, promoter methylation is usually repressive, silencing the adjacent gene across diverse taxa (Klughammer et al., 2023; Niederhuth et al., 2016).

In contrast to its typically repressive impact on TEs, promoters and regulatory regions, DNA methylation appears to serve a different function in coding sequences, although our understanding of its precise function remains incomplete. Gene‐body methylation (gbM), which characteristically occurs exclusively in the CpG context, is present in the coding sequences of many constitutively expressed genes in plants (Muyle et al., 2022; Niederhuth et al., 2016) and animals (Lewis et al., 2020; Männer et al., 2021; Sarkies, 2022), yet is not present across all genes in a genome nor across all species in a taxon (e.g. Dixon & Matz, 2022), suggesting redundancy in its function. In plants (Muyle et al., 2022) and some invertebrates (Olson & Roberts, 2014; Wang et al., 2013), gbM positively correlates with constitutive gene expression, suggesting a role in transcriptional regulation. It may also contribute to the definition of exonic boundaries, increasing splicing accuracy in animals (Lev‐Maor et al., 2015; Shayevitch et al., 2018), although this effect is unclear in plants (Bewick et al., 2016; Horvath et al., 2019). High gbM and low promoter methylation may represent alternative forms of gene expression regulation, with vertebrates adopting the latter as a means of controlling tissue‐specific expression (Keller et al., 2016), and more generally, the taxonomic diversity in the presence and functioning of these two distinct forms of methylation underscores how diverse epigenetic regulation is closely tied to evolutionary change.

### 2.2 Resetting and inheritance of epigenetic marks across generations

Epigenetic modifications can be stably inherited across cell divisions (Zhang & Sirard, 2021), but transmission fidelity is generally higher during mitotic than meiotic divisions due to epigenetic reprogramming during meiosis (Kawashima & Berger, 2014), which resets many DNA methylation marks (Becker et al., 2011; Feng et al., 2010). Epigenetic resetting is particularly pronounced in mammalian vertebrates, while patterns of DNA methylation may be better preserved during germ cell differentiation in other taxa, such as fish (Ortega‐Recalde & Hore, 2019). In plants, epigenetic reprogramming is primarily observed in the male germline and in the CHG and CHH sequence contexts (Calarco et al., 2012; Wibowo et al., 2016). Across organisms, the first stage of resetting involves active enzyme‐catalyzed removal of DNA methylation marks (Gallego‐Bartolomé, 2020; Gong & Zhu, 2011), followed by a re‐establishment of DNA methylation marks guided by ncRNAs (Fallet et al., 2023). In plants, small interfering RNAs (siRNAs), generated from active TEs, enter the pollen and the fertilized egg, where they guide the establishment of epigenetic marks (Dunoyer et al., 2013; Slotkin et al., 2009), similar to the piwi‐interacting RNAs (piRNAs) that induce epigenetic modifications from one allele to the other in Drosophila (De Vanssay et al., 2012). In some cases, methylation marks are not renewed in newly synthesized DNA strands after replication, resulting in a gradual disappearance of the methylation status (i.e. passive demethylation).

Variation in the extent of resetting means that DNA methylation can either be reset across sexual generations, or persist across one (intergenerational inheritance) (Boyko et al., 2010; Verhoeven et al., 2010) or multiple generations (transgenerational inheritance) (Johannes et al., 2009; Ou et al., 2012). Intergenerational epigenetic inheritance (or parental effects) can also arise from embryonic reserves, such as hormones, nutrients and ncRNAs, that persist into the first offspring generation (Badyaev & Uller, 2009). In contrast, transgenerational epigenetic inheritance involves the transmission of epigenetic variants that cannot be attributed to the direct effects of the original trigger (Fitz‐James & Cavalli, 2022). In the case of environmental triggers, transgenerational epigenetic inheritance could thus represent a vector of stress memories across generations, with a stronger potential to contribute towards evolutionary processes.

The transmission of DNA methylation across mitotic divisions can vary depending on the sequence context. Plant‐specific methylation in CHG and CHH sequence contexts is less stable than CpG methylation, where the presence of a complementary cytosine on the other DNA strand allows for the reliable guidance of DNA methylation in the newly synthesized daughter strands (Harrison et al., 2016). The persistence of DNA methylation across generations also seems to depend on the reproductive mode (for a thorough review, see Anastasiadi et al., 2021). Clonally reproducing organisms, which lack meiosis, may exhibit less epigenetic reprogramming and thus more faithful transmission of DNA methylation across clonal generations compared to sexually reproducing species (Latzel et al., 2016; Latzel & Klimešová, 2010; Verhoeven & Preite, 2014). In both plants and animals, this extensive inheritance of DNA methylation could potentially account for the ecological success of clonal species, which often display broad natural distributions even in the absence of substantial genetic variation (Dodd & Douhovnikoff, 2016; Vogt, 2022). However, some epigenetic reprogramming associated with development is expected even across clonal generations, and definitive evidence for the higher stability of epigenetic modifications across clonal generations compared to sexual generations remains elusive.

Beyond DNA methylation, most histone modifications appear to be stable for no longer than a few days (reviewed in Cedar & Bergman, 2009 and in Lämke & Bäurle, 2017), although recent studies in the fission yeast *Schizosaccharomyces pombe* (Audergon et al., 2015) and the nematode *Caenorhabditis elegans* (Klosin et al., 2017; Lee et al., 2019) have revealed that various methylated marks of lysine 9 of histone 3 (H3K9me, H3K9me2 and H3K9me3) can be transgenerationally stable for more than 10 generations. Following DNA replication, modified histones are randomly distributed among the parental and newly synthesized DNA strands (Alabert et al., 2015). These then recruit histone‐modifying proteins that copy the modifications to the unmodified histones (Moazed, 2011), leaving the possibility for histone modifications to be maintained through cell divisions and inherited across generations if they remain stable. ncRNAs also play a role in epigenetic inheritance and can be transferred intergenerationally (Bilichak et al., 2015; Morgado et al., 2017), provided they become part of the cellular matrix of egg or sperm cells. Among all epigenetic mechanisms, ncRNAs are the only mobile modifications moving within and across cell barriers (Creemers et al., 2012; Dorval et al., 2013), and indeed, the transfer of epigenetic information from somatic tissue to the germline via miRNAs has been demonstrated in human cell culture and *C. elegans* (Devanapally et al., 2015; Sharma, 2017; Szyf, 2015), suggesting that parental ncRNAs, though short‐lived themselves, may help establish offspring methylation patterns that last several generations (Beck et al., 2021). Whatever mechanism is being considered, the persistence of epigenetic variation across multiple generations is likely to depend upon the system in question and the many extrinsic (environmental) factors at play (Anastasiadi et al., 2021). For example, the presence of transgenerational inheritance of histone marks in *S. pombe* and *C. elegans* may compensate for the lack of DNA methylation in these organisms and may be associated with specific environmental stresses such as temperature (Klosin et al., 2017).

## 3 THE INTERDEPENDENCE OF GENOME AND EPIGENOME

As we have seen, the epigenome is a rich source of variation in addition to that of the genome, with the potential to be transmitted transgenerationally under certain conditions. However, it remains open to debate whether the epigenome simply represents a regulatory extension of the genome or whether the combination of environmental sensitivity, heritability and functional consequences confers upon it additional evolutionary roles. In order to answer this question, it is imperative to first disentangle the intricate interactions and interdependence between the genome and epigenome.

### 3.1 The genetic and environmental determinants of natural epigenetic variation

Determining the relative contributions of the underlying genetic structure and environmental factors to natural population epigenetic variation is increasingly feasible with population genetic approaches. Recent comprehensive studies in the established and emerging plant models *A. thaliana* (Kawakatsu et al., 2016), *Thlaspi arvense* (field pennycress) (Galanti et al., 2022) and *Fragaria vesca* (woodland strawberry) (Sammarco et al., 2024) suggest that, at least in these herbaceous species, most epigenetic variation observed between natural populations is explained either by *cis* or *trans* genetic modifiers (Figure 1). The power of model systems such as *A. thaliana* is clear, as the associations of >1000 genomes, methylomes and transcriptomes can be leveraged to investigate interactions between the epigenome and genome (Kawakatsu et al., 2016) and to identify specific genetic polymorphisms that regulate variation in DNA methylation across populations (Sasaki et al., 2019). *Cis* modifiers are often associated with the presence or absence of nearby TE insertions, as their heavily methylated states tend to spread over nearby regions (Martin et al., 2009; Quadrana et al., 2016), while *trans* modifiers comprise a wide range of well‐known epigenetic regulators such as methyltransferases or genes involved in the RNA‐directed DNA methylation pathway (Dubin et al., 2015; Galanti et al., 2022; Kawakatsu et al., 2016; Sammarco et al., 2024; Sasaki et al., 2019, 2022). Less is known about the extent to which genetic and epigenetic variation are linked in animals (Hu & Barrett, 2017), but a recent study of the Olympia oyster (*Ostrea lurida*) found that roughly a third of methylation variation is associated with genotypic differences (Silliman et al., 2023). This finding implies that a substantial portion of the variation remains unaccounted for, but it also underscores the significant influence of the genome on epigenetic variation in animals, which aligns with the evidence gathered in plants.

**FIGURE 2:**
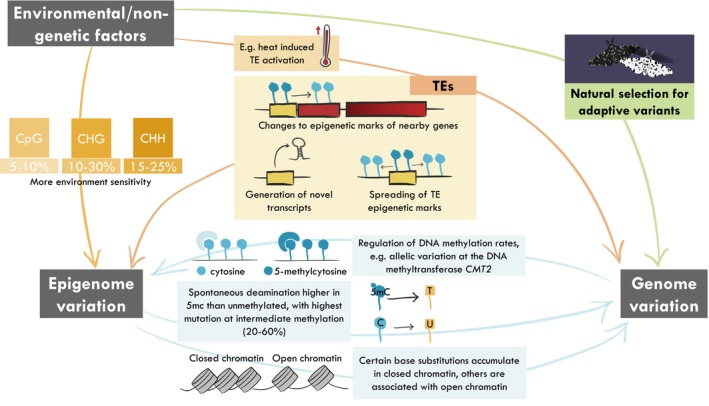
The genome and epigenome are interdependent, with changes in one influencing the other (blue arrows). The environment can affect both the former through direct effects on epigenetic processes like methylation (orange arrow) and the latter through natural selection for adapted genetic variants (green arrow). Transposable Elements (TEs) fill a critical position at the interface of all three factors, as they are able to translate environmental or stochastic variation into both epigenomic and genomic variation (red arrows).

While a large fraction of epigenetic variation, at least in plants, appears to be dependent on the genome, a smaller yet significant fraction is often found to be better explained by the environment than the underlying genetic structure. This result has been observed for DNA methylation across taxonomically diverse natural populations of plants (De Kort et al., 2020; Jueterbock et al., 2020; Martinelli et al., 2021) and animals (Aagaard et al., 2022; Chapelle & Silvestre, 2022; Johnson & Kelly, 2020). In two of the most recent comprehensive population epigenetic studies of plants, most epigenetic variation was found to be genetic in origin (~90%–95% in CG, ~70%–90% in CHG and 55%–65% in CHH), but the environment still explained a significant fraction of the observed variation (~5%–10% in CG, 10%–30% in CHG and 15%–25% in CHH; Figure 2) (Galanti et al., 2022; Sammarco et al., 2024). These studies showed that the methylation sequence context was an important determinant of environmental sensitivity. The proportion of epigenetic variation under environmental control is higher in the CHG and CHH sequence contexts than the CpG sequence context, a result in accordance with CHG and CHH methylation being more responsive to stress (Gáspár et al., 2019; and reviewed in Liu & He, 2020). Among animals, variation in the environmental sensitivity of methylation also exists, and probably occupies a continuum from highly stable marks to environmentally sensitive ones, as shown in *Gasterosteus aculeatus* by Heckwolf et al. (2020). Thus, while genetic variation appears to be the primary contributor to epigenetic variation in both plant and animal populations, current evidence indicates that environmental factors also significantly influence epigenetic variation. This underscores the potentially pivotal role of epigenetic variation in shaping adaptation and evolution independently of genetic variation.

Still, evidence for the role of the environment in the induction of heritable epigenetic variation remains scarce. Although extensive DNA methylation changes (notably over TE sequences) can be observed following environmental change such as mild drought or salt stress in *A*. *thaliana*, only a minute and stochastic fraction were found to be heritable transgenerationally (Ganguly et al., 2017; Van Dooren et al., 2020; Wibowo et al., 2016). As a result, part of the environmentally associated heritable DNA methylation variation observed in natural populations may be explained by environmentally associated but unaccounted‐for components of the genetic architecture rather than by direct environmental effects. Indeed, in *A. thaliana*, CHH methylation variation at TEs is largely associated with allelic variation at the DNA methyltransferase *CMT2* (Figure 2), whose distribution follows environmental clines, presumably because of local selection, even though the phenotypes associated remain elusive (Dubin et al., 2015). More generally, when environmental effects are weak, it can be difficult to associate the remaining epigenetic variation with the underlying genetic structure, leaving a significant fraction unexplained (e.g. as for *Parus major*; Sepers et al., 2023).

Although the extent of inheritance of environmentally induced epigenetic variation is still debated, unambiguous evidence from mutation accumulation lines demonstrates that epimutations (i.e. heritable epigenetic changes) can occur spontaneously and be stably inherited transgenerationally (Becker et al., 2011; Denkena et al., 2021). Moreover, the rate at which spontaneous epimutations occur is orders of magnitude higher than that of single nucleotide mutations or other DNA sequence variants, ruling out underlying genetic effects (Yao et al., 2023). Regions of the genome where epimutations accumulate in a clock‐like manner, independently of genetic backgrounds or environmental conditions, have been identified in *A. thaliana* and the clonal seagrass *Zostera marina* and used to recapitulate phylogenies with high resolution (Yao et al., 2023). Moreover, this study also highlighted how, in other regions of the genome, epimutation rates vary significantly across different environments. Thus, epigenetic differences observed between natural populations (that may or may not have diverged in their environments) may also result from fast‐occurring stochastic and stably inherited epimutations, whose rate of occurrence could be influenced by the environment, rather than from epigenetic changes that are directly induced by the environment. Supporting this conclusion, De Kort et al. (2020) observed that drought stress induced no immediate change in DNA methylation in woodland strawberry, suggesting that the drought‐associated DNA methylation differences observed between wild isolates reflect population history rather than immediate plastic responses to the environment (De Kort et al., 2020). Thus, while the possibility of environmentally induced epimutations that persist across generations remains uncertain, the role of the environment in selecting and modulating the rate of occurrence of random epimutations can be easily envisioned.

### 3.2 Epigenetic factors influencing mutation rates

So far, we have discussed how genetic variation influences epigenetic variation, but it is important to note that the reverse relationship holds true as well. Several types of epigenetic marks have been demonstrated to impact mutation rates, both positively and negatively. DNA methylation in particular has been associated with increased mutation rates, presumably due to 5‐methylcytosine being more prone to spontaneous deamination compared to unmethylated cytosines (reviewed in Danchin et al., 2019 and Pfeifer, 2006; Figure 2). Recent studies have also shown that the highest mutation rates occur at intermediate levels of methylation (20%–60%), while higher levels of methylation could actually protect the sites from mutation (Ord et al., 2023; Venney et al., 2022). Similarly, certain types of DNA methylation, such as 5‐hydroxymethylcytosine (5hmC), may be less efficiently deaminated and show reduced mutation frequency (reviewed in Tomkova & Schuster‐Böckler, 2018). In mammals, 5hmC is particularly elevated in actively transcribed genes, which would benefit from lower mutation rates (Ficz et al., 2011; Mellén et al., 2012).

Chromatin accessibility has also been shown to have an impact on mutation rates; however, the impact depends on the type of mutation: base substitutions accumulate in closed chromatin regions (Adar et al., 2016), while mutational states associated with high rates of insertions and deletions (indels) and substitutions have been found to be enriched in certain open chromatin regions (reviewed in Makova & Hardison, 2015; Figure 2). Additionally, the histone mark H3K4me1 was associated with genome stability and lower mutation rates in several studies (Ha et al., 2017; Herbette et al., 2017). There may also be interactive effects between chromatin states, histone modifications and DNA methylation, although collinearity between different epigenetic modifications can make it challenging to disentangle their effects (Glastad et al., 2016).

Overall, there is clear evidence that epigenetic marks affect genetic mutation rates. A recent study showed that GC content, H3K4me1 and many other epigenetic marks could accurately predict mutation bias occurring across the *A. thaliana* genome (Monroe et al., 2022, 2023). Monroe and colleagues also showed a reduced mutation rate in gene bodies, particularly in essential genes (Monroe et al., 2022, 2023). Although there has been some debate about whether their results might be biased by sequencing artefacts (Liu & Zhang, 2022; Monroe et al., 2023; Wang et al., 2023), taken with the other aforementioned results, these findings strengthen the argument that epigenetic marks play an important role in determining genetic mutation rates.

### 3.3 TEs at the crossroads between genome, epigenome and environment

Through their ability to translate epigenetic and environmental variation into new genetic variation in the form of transposition events, TEs represent unique sensors linking the genome, epigenome and environment (Figure 2). In plants, experiments in *A. thaliana* have shown that impairing the pathways responsible for the epigenetic silencing of TEs can trigger transposition (Quadrana et al., 2019; Tsukahara et al., 2009). However, the reactivation of some TEs also requires additional exposure to an environmental stressor (Baduel et al., 2021; Thieme et al., 2022; e.g. heat stress, Figure 2), highlighting how TEs can act as integrators of epigenetic and environmental signals into genetic variation. These unique properties can then be co‐opted for gene regulation, as illustrated by the new layer of stress regulation brought to a major flowering time regulator by an insertion of a stress‐sensitive TE (Quadrana et al., 2019), a recurrent event throughout Brassicaceae species at this locus (Quadrana, 2020). Remarkably, this co‐option process may have occurred recurrently throughout the evolution of eukaryote genomes, as many TE fragments marked by the tri‐methylation of lysine 27 of histone 3 (H3K27me3) play the role of regulatory regions (Hisanaga et al., 2023). Furthermore, evidence from distantly related eukaryotes suggests the ancestral function of this histone mark, which is associated with gene silencing in flowering plants and animals, was to silence TEs (Hisanaga et al., 2023). TE silencing is associated with H3K9me3 in most eukaryotes (reviewed in Shlyueva et al., 2014 and Ninova et al., 2019), and its appearance in a relatively basal ancestor appears to have coincided with the estimated invasion of eukaryotic genomes by retrotransposons (Kabi & Filion, 2021), highlighting a potentially important role that TEs may have played at the interface between genome and epigenome in deep evolutionary history.

In addition to being major mutagens, TEs also have the potential to affect local epigenetic landscapes and potentially alter nearby gene expression (Coronado‐Zamora & González, 2023 and reviewed in Choi & Lee, 2020; and in Kelleher et al., 2020). In *A. thaliana*, DNA methylation of TE sequences can spread to previously unmethylated regions upon insertion (Hollister & Gaut, 2009; Quadrana et al., 2016), spreading them several hundred to several thousand base pairs. However, in the grass *Brachypodium*, DNA methylation around TEs spreads over shorter distances compared to *A. thaliana*, and in only a small fraction of insertions did TE methylation influence the expression of adjacent genes (Wyler et al., 2020). Thus, while being major mutagens, TEs can also have a profound impact on the local epigenetic landscapes and gene expression. This influence varies across different species and is dependent on the extent of the spread of epigenetic marks around the TEs.

The epigenetic impact of TE insertions over longer evolutionary timescales can be revealed through comparative studies of TE abundance and associated epigenetic marks in closely related species. For example, TE‐mediated local enrichment of repressive marks varied substantially among (and even within) six *D. melanogaster* subgroup species (Huang et al., 2022). Notably, higher expression of suppressor of position‐effect variegation [Su(var)] genes promoted a repressive chromatin environment, potentially leading to reduced fitness and stronger selection against TEs that induce epigenetic effects (Huang et al., 2022). On the other hand, the presence of TEs near promoters can lead to changes to the epigenomic regulatory landscape and the co‐option of TEs, as seen in closely related *Heliconius* butterfly species, where novel TE insertions and associated differences in chromatin accessibility shape genome evolution (Lewis & Reed, 2019; Ruggieri et al., 2022). In the same vein, young TEs showed substantial methylome divergence among closely related Lake Malawi cichlids, potentially facilitating the adaptive radiation of this group (Vernaz et al., 2021). At even broader scales, the proportion of repetitive regions in the genome is likely to be a key determinant of methylation, as seen in a comparison of 40 diverse fungi species (Bewick et al., 2019), and epigenetic control of TEs could be critical in explaining the emergence of many novel regulatory mechanisms in vertebrates (reviewed in Almeida et al., 2022). In conclusion, the presence of TEs can significantly shape the epigenetic landscape and modulate gene regulation, potentially leading to evolutionary advantages or disadvantages. However, the relationship between TEs and epigenetics is complex and influenced by many factors, including the genomic architecture of the host, the genomic locations of the TE insertions and the specific characteristics of the TEs themselves.

## 4 FUNCTIONAL CONSEQUENCES OF POPULATION EPIGENETIC VARIATION

**FIGURE 3:**
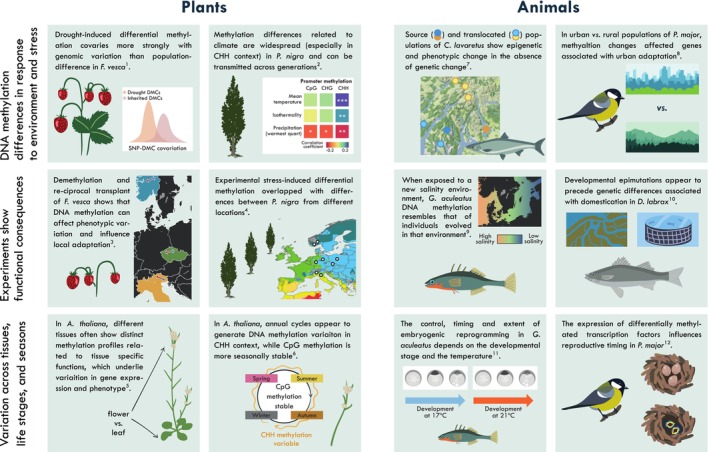
Established and emerging plant and animal model systems are helping to reveal the extent of natural variation in the epigenome and to demonstrate its functional consequences for phenotypic evolution. References: [1] De Kort et al. (2022), [2] Díez Rodríguez et al. (2022), [3] Sammarco et al. (2022), [4] Peña‐Ponton et al. (2022), [5] Wibowo et al. (2022), [6] Ito et al. (2019), [7] Crotti et al. (2021), [8] Caizergues et al. (2022), [9] Heckwolf et al. (2020), [10] Anastasiadi and Piferrer (2019), [11] Fellous et al. (2022), [12] Lindner et al. (2021).

As we have seen, epigenomic variation, while tightly linked to genomic factors, may also arise spontaneously or be environmentally induced. If transgenerationally inherited, these epigenomic changes could potentially influence adaptive evolution, but for this to hold true, such changes should also entail functional phenotypic consequences upon which selection can act (Bonduriansky et al., 2012). It has recently been shown that stable epimutations can have phenotypic effects even in the absence of genetic variation (Wibowo et al., 2022), and while linking epigenetic variation and natural phenotypic variation remains challenging, a number of recent studies using experimental and comparative approaches have shed light on some of the functional roles of epigenomic variation and its associated phenotypic effects (Figure 3).

### 4.1 DNA methylation differences in response to environment and stress

Disentangling genetic and epigenetic contributions to phenotypic variation in natural populations remains difficult. However, evidence from plants indicates that DNA methylation variation is sometimes a better predictor of functional trait variation than genetic variation, especially when the latter is very low (Herrera et al., 2017; Wang et al., 2020). Associations between DNA methylation levels and key traits across large taxonomic scales could highlight evolutionary transitions, as observed in a comparison of 279 angiosperms, in which woody species showed lower DNA methylation independently of phylogeny (Alonso et al., 2019). At the population level, temperature‐dependent DNA methylation of *F. vesca* influenced flowering time and the number of growth points in certain ecotypes, as well as being stably transmitted across clonal generations (Zhang et al., 2023). In the same species, drought‐induced methylation changes associated with stress‐responsive genes correlated strongly with population genomic variation, highlighting the link between environmentally sensitive methylation and adaptive potential (De Kort et al., 2022; Figure 3). Among animals and other taxa, less is known about how population epigenetic variation and phenotype are linked (Hu & Barrett, 2017), in part because DNA methylation is absent in the commonly used lab animals *D. melanogaster* and *C. elegans* (Yi, 2017). As our understanding of epigenomic effects in a broad variety of organisms grows, experimental approaches are being used to highlight environmental sensitivity in the epigenome and potential functional consequences.

Epigenetic change following exposure to novel environmental stresses can help to highlight the sensitivity of the epigenome in natural populations, and while often these stresses have been associated with negative phenotypic effects (Vandegehuchte & Janssen, 2014), in some cases, they appear to provide enhanced stress tolerance to progeny (Ou et al., 2012). Fish reared in novel environments acquire predictable changes in DNA methylation in potentially functionally important regions of the genome, a result that may be repeatable across populations (Sävilammi et al., 2021), leading to genome‐wide patterns of DNA methylation that resemble those of the individuals that evolved in that environment (Artemov et al., 2017; Heckwolf et al., 2020; Figure 3). Furthermore, these epigenomic changes may also result in clear phenotypic differences, as shown in European whitefish (*Coregonus lavaretus*) populations translocated to novel lake environments, which accumulated many morphological and methylation differences over 30 years in the absence of strong genetic differences (Crotti et al., 2021; Figure 3). Urban environments may be a particularly fruitful setting for investigating the role of the epigenome in rapid adaptation, as they represent evolutionary recent novel environments that differ substantially from natural environments in terms of food, nutrient availability/quality and pollution levels (Watson et al., 2021). For example, urban populations of *Parus major* show differential DNA methylation compared with their rural counterparts (Caizergues et al., 2022; Watson et al., 2021), with many of the differentially methylated regions close to genes with stress response and aggression functions, which are predicted to respond in urban‐adapted populations (Caizergues et al., 2022; Figure 3).

Evidence is thus growing that changes in DNA methylation may facilitate adaptation to novel environments. In particular, it may underlie the success of invasive and clonal species, compensating for the low genetic variation (Carneiro & Lyko, 2020; Mounger et al., 2021) and potentially explaining phenotypic change in the absence of genetic differentiation (Thorson et al., 2017). Introduction to novel stressful environments may lead to reductions in DNA methylation, potentially enhancing phenotypic plasticity when it is most required to overcome genetic barriers such as bottlenecks or inbreeding (Ardura et al., 2017, 2018). Furthermore, it is not only invading species that may show responses upon exposure to novel stressful environments; the invasive species itself may serve as a biotic stressor, inducing reductions in DNA methylation and associated phenotypic differences in native species (Schrey et al., 2016).

### 4.2 Experiments reveal functional consequences of epigenetic variation

Beyond studies of natural populations, common garden and controlled laboratory experiments provide an important complementary approach to determine the respective contributions of genetic, environmental factors to the epigenome, and how this in turn generates phenotypic variation. In plants, the phenotypic effects of heritable variation in DNA methylation have been most clearly demonstrated using epigenetic recombinant inbred lines (so‐called epiRILs) in *A. thaliana* that segregate experimentally induced loss of DNA methylation in the absence of DNA sequence variants, notably the mutation used to induce them (Johannes et al., 2009). These lines were propagated by single‐seed descent for several generations and then used to map the epigenetic basis of complex traits, such as flowering time or root length (Cortijo et al., 2014). Recent efforts to re‐sequence the genome and the methylome of the epiRILs have provided further evidence that the inheritance of DNA methylation loss in these lines is truly independent from any DNA sequence changes (Kakoulidou et al., 2024; Quadrana et al., 2019; Zhang et al., 2021). In non‐model systems, where such genetic tricks cannot be used, the functional impact of DNA methylation loss can be nonetheless investigated using a demethylation agent, such as 5‐azacytidine or zebularine. Effects of demethylation can be beneficial, such as increased biomass (Vergeer et al., 2012), or increased salt tolerance (Song et al., 2022). Sammarco et al. (2022) used demethylation and reciprocal transplant experiments to show that in some *F. vesca* populations DNA methylation affected phenotypic variation and played a role in local adaptation (Figure 3). Such a role was, however, contingent on the population in question and environment being tested. Furthermore, Herden et al. (2019) showed that for 12 different ruderal species, demethylation generally had neutral or negative effects on aboveground biomass, with an increase in biomass only observed in a single species.

Populations that are undergoing domestication are not only important for understanding the consequences of epigenetic variation in applied systems but can also be particularly convenient models for investigating functional epigenetic responses. For example, in the absence of genetic differentiation, captive breeding produced strong changes in the DNA methylation of coho salmon (*Oncorhynchus kisutch*) near genes likely to be involved in seawater acclimation and neuromuscular processes (Le Luyer et al., 2017). Similarly, in the Atlantic salmon (*Salmo salar*), a single generation of captive breeding generated transgenerationally stable epigenetic changes near genes with functions associated with domestication, such as aggression and olfaction (Rodriguez Barreto et al., 2019). Developmental epimutations appear to be key in the domestication of the European sea bass (*Dicentrarchus labrax*), and precede genetic differences (Anastasiadi & Piferrer, 2019; Figure 3). Among plants, artificially propagated clonal species can also offer an informative perspective, such as poplar (*Populus*), in which transcriptomic responses to drought mirror global differences in genome‐wide DNA methylation (Raj et al., 2011). The *Populus nigra cv “Italica”* clone has a global distribution and environmentally sensitive DNA methylation, with experimental stress‐inducing differential methylation in stress‐responsive genes that also differ between populations (Díez Rodríguez et al., 2022; Peña‐Ponton et al., 2022; Vanden Broeck et al., 2018; Figure 3).

### 4.3 Epigenomes are temporally dynamic across life stages and seasons

The epigenome plays a fundamental role in development, cell differentiation and organogenesis. In both animals (Blake et al., 2020; Watson et al., 2021) and plants (D'Amico‐Willman et al., 2022; Vining et al., 2012), different tissues often show distinct methylation profiles related to tissue specific functions, that can underlie variation in gene expression and phenotype (Wibowo et al., 2022; Figure 3). Furthermore, differences in DNA methylation between tissues and life stages are generally more marked than population or species differences (Blake et al., 2020; Liu et al., 2022; Zhou et al., 2022). Embryogenic epigenetic differences and correlated expression may be key to explaining the development of distinct ecomorphs (Matlosz et al., 2022) and highlight the developmental pathways involved in the evolution of different ontogenetic strategies, as shown by Davidson et al. (2022) in their comparison of lecithotrophic (directly developing) and planktotrophic sea urchins. In animals, the epigenome appears to be more sensitive to environmental variation during the earliest life stages (Anastasiadi et al., 2017; Baldanzi et al., 2022), which may extend to later developmental stages in some taxa, such as birds (Watson et al., 2019). Thus, environmental changes experienced during early life may alter patterns of epigenetic reprogramming, leading to functionally important changes in gene expression during this critical point in phenotypic development (Fellous et al., 2022; Figure 3).

The epigenome has also been predicted to vary according to temporal patterns, for example, promoting seasonal changes in phenotypes. DNA methylation appears to play a role in regulating seasonal timing in birds (Viitaniemi et al., 2019), with differentially methylated transcription factors influencing reproductive timing (Lindner et al., 2021; Figure 3), and the methylation status of intergenerationally inherited *clock* genes correlating with breeding date (Saino et al., 2019). In *A. thaliana*, annual cycles appear to generate DNA methylation variation, at least in the CHH context, while CpG methylation appears more seasonally stable (Ito et al., 2019). While circadian epigenomic cycles have been observed in laboratory plant (Perales & Más, 2007) and mammal (Azzi et al., 2014) systems, changes in natural populations appear weak, at least in terms of DNA methylation (Diao et al., 2017). Given our improved understanding of the changes in histone and chromatin underlying circadian rhythms (Xiong et al., 2022), experiments that measure these marks may be more appropriate for identifying temporal and cyclical changes to the epigenome.

## 5 CONCLUSIONS AND PERSPECTIVES

Ten years ago, Heard and Martienssen (2014) surmised that the role of transgenerational inheritance of epigenetic characters outside of plants remained equivocal, and that for all taxa, the extent to which environmentally induced effects were transmitted across generations was uncertain. Since then, the study of epigenomics in many natural plant and animal populations has progressed rapidly, revealing a remarkable amount of population epigenetic variation (see reviews by Chapelle & Silvestre, 2022; Husby, 2022). What has been less clear until now is the evolutionary importance of this variation: whether epigenetic effects are independent of the genome, and the link between environmental and epigenomic variation. Increasing evidence points towards epigenetic effects that are largely determined by genomic variation, especially in plants, but with a measurable fraction environmentally induced (Galanti et al., 2022; Sammarco et al., 2024). The evidence that stable epigenetic changes are directly induced by the environment remains weak (De Kort et al., 2020), suggesting that epigenetic differences between populations may be more stochastic in origin. Nonetheless, functionally relevant epigenetic variants may potentially be transmitted across generations if environmental manipulation is maintained (Heckwolf et al., 2020). Given that variation in the epigenome can itself affect mutation rates (Venney et al., 2022), notably through TE mobilization (Quadrana et al., 2019), and is linked to nucleotide diversity (Ord et al., 2023), it remains possible that environmental sensitivity in the epigenome plays a key role in shaping evolutionary processes, especially if mutations occur directly at the locus containing the epimutation, a region that may be functionally important in the response to the environmental stimulus (Sabarís et al., 2023). Determining the evolutionary consequences of environmental and stochastic epigenomic variation will require research combining high resolution population epigenetic information with multigenerational experimental manipulation.

While some features of the epigenome, such as gbM, appear to be nearly universal across eukaryotic taxa, plants and animals display important differences, most likely due to the timing of germline separation. The later separation of germline and soma in plants (Grossniklaus, 2011) may grant plants greater capacity to epigenetically react to environmental changes and transmit them to their offspring, helping them to adapt to changing environmental conditions (Jablonka & Lamb, 2015). Furthermore, CHG and CHH methylation, which are more environmentally sensitive than CpG methylation (Liu & He, 2020), are more commonplace in plants than animals, which may also help to explain the plasticity of the plant epigenome. An important advance of recent years is that technological improvements in sequencing and computing now allow the characterization of epigenomic variation across tens (Lewis et al., 2020) and even hundreds (Klughammer et al., 2023) of species. Such approaches have revealed, for example, how hypermethylation has evolved independently in some arthropods (Lewis et al., 2020) and that specific DNA sequence compositions can be highly predictive of methylation across broad metazoan lineages (Klughammer et al., 2023). Comparative approaches that include both within‐ and between‐species comparisons will be enormously helpful in generalizing our understanding of interactions between genome and epigenome, as will new bioinformatic approaches that can leverage existing sequence data to make novel inferences (Ord et al., 2023). Future experimental work will benefit from using functional genomic tools, with several CRISPR‐based epigenetic editing strategies now functioning in vivo (Nakamura et al., 2021). While these are currently limited to model systems like *A. thaliana* (Chen et al., 2024), other functional genomic tools such as RNAi are more readily applicable to non‐model systems (Gudmunds et al., 2022). Furthermore, the development of CRISPR techniques for homology‐directed repair could be of particular benefit in the validation of quantitative trait loci under ecologically realistic conditions (Gudmunds et al., 2022).

Other important goals in the field include further research efforts to integrate other epigenetic factors, including histone modifications, chromatin accessibility and ncRNAs, into our understanding of epigenomics in natural populations. These factors serve different but interdependent roles, and recent studies that account for multiple levels reveal important interactions between them (Sun et al., 2021), and recent technological advances should make measuring multiple levels simultaneously increasingly feasible (Lee et al., 2020; Lhoumaud et al., 2019). It is also important to expand the use of epigenomic information in conservation biology (Rey et al., 2020), especially in systems where environmental sensitivity in the genome can impact phenotypes and population viability (Lockley & Eizaguirre, 2021), and in understanding the genetic and epigenetic basis of invasiveness (Marin et al., 2020). Finally, it should be an important goal to incorporate our understanding of epigenomic variation into eco‐evolutionary models, as is the case for genomic data (Waldvogel et al., 2020), which could allow epigenomic factors to aid in predictions of how natural populations will respond to global change.

## CONFLICT OF INTEREST STATEMENT

The authors have no conflict of interest to declare.

## Data Availability

Data sharing not applicable to this article as no datasets were generated or analyzed during the current study.
